# Prediction of Asthma Hospitalizations for the Common Cold Using Google Trends: Infodemiology Study

**DOI:** 10.2196/27044

**Published:** 2021-07-06

**Authors:** Bernardo Sousa-Pinto, Jaana I Halonen, Aram Antó, Vesa Jormanainen, Wienczyslawa Czarlewski, Anna Bedbrook, Nikolaos G Papadopoulos, Alberto Freitas, Tari Haahtela, Josep M Antó, João Almeida Fonseca, Jean Bousquet

**Affiliations:** 1 MEDCIDS – Department of Community Medicine, Information and Health Decision Sciences; Faculty of Medicine, University of Porto Porto Portugal; 2 CINTESIS – Center for Health Technology and Services Research; University of Porto Porto Portugal; 3 Finnish Institute for Health and Welfare (THL) Helsinki Finland; 4 MASK-air Montpellier France; 5 Medical Consulting Czarlewski Levallois France; 6 MACVIA-France Montpellier France; 7 Allergy Department, 2nd Pediatric Clinic, Athens General Children’s Hospital “P&A Kyriakou”, University of Athens Athens Greece; 8 Division of Infection, Immunity & Respiratory Medicine, Royal Manchester Children’s Hospital, University of Manchester Manchester United Kingdom; 9 Skin and Allergy Hospital, Helsinki University Hospital, and University of Helsinki Helsinki Finland; 10 ISGlobal, Barcelona Institute for Global Health Barcelona Spain; 11 Universitat Pompeu Fabra Barcelona Spain; 12 IMIM - Hospital del Mar Medical Research Institute Barcelona Spain; 13 CIBER Epidemiología y Salud Pública - CIBERESP Barcelona Spain; 14 Charité, Universitätsmedizin Berlin, Humboldt-Universität zu Berlin Berlin Germany; 15 Comprehensive Allergy Center, Department of Dermatology and Allergy, Berlin Institute of Health Berlin Germany; 16 University Hospital Montpellier France

**Keywords:** asthma, common cold, Google Trends, hospitalizations, time series analysis, mobile phone

## Abstract

**Background:**

In contrast to air pollution and pollen exposure, data on the occurrence of the common cold are difficult to incorporate in models predicting asthma hospitalizations.

**Objective:**

This study aims to assess whether web-based searches on *common cold* would correlate with and help to predict asthma hospitalizations.

**Methods:**

We analyzed all hospitalizations with a main diagnosis of asthma occurring in 5 different countries (Portugal, Spain, Finland, Norway, and Brazil) for a period of approximately 5 years (January 1, 2012-December 17, 2016). Data on web-based searches on *common cold* were retrieved from Google Trends (GT) using the *pseudo-influenza syndrome* topic and local language search terms for *common cold* for the same countries and periods. We applied time series analysis methods to estimate the correlation between GT and hospitalization data. In addition, we built autoregressive models to forecast the weekly number of asthma hospitalizations for a period of 1 year (June 2015-June 2016) based on admissions and GT data from the 3 previous years.

**Results:**

In time series analyses, GT data on *common cold* displayed strong correlations with asthma hospitalizations occurring in Portugal (correlation coefficients ranging from 0.63 to 0.73), Spain (*ρ*=0.82-0.84), and Brazil (*ρ*=0.77-0.83) and moderate correlations with those occurring in Norway (*ρ*=0.32-0.35) and Finland (*ρ*=0.44-0.47). Similar patterns were observed in the correlation between forecasted and observed asthma hospitalizations from June 2015 to June 2016, with the number of forecasted hospitalizations differing on average between 12% (Spain) and 33% (Norway) from observed hospitalizations.

**Conclusions:**

Common cold–related web-based searches display moderate-to-strong correlations with asthma hospitalizations and may be useful in forecasting them.

## Introduction

### Background

Asthma poses a substantial burden on health care, with hospitalizations being one of the main drivers of asthma-related costs [1]. The prediction of asthma hospitalization patterns may take into account major risk factors for asthma exacerbations, such as occurrence of the common cold (most often due to rhinovirus infections) [2-4], air pollution, and pollen exposure. However, although air pollution and pollen peaks can be measured (allowing for alert systems to be developed), data on rhinovirus infections are more difficult to obtain and thus to be incorporated into prediction models.

Infodemiology data open new possibilities for the development of models predicting asthma hospitalizations. Infodemiology is defined as “the science of distribution and determinants of information in an electronic medium, specifically the Internet, or in a population, with the ultimate aim to inform public health and public policy” [5,6]. Infodemiology comprises *supply-based* and *demand-based* approaches, with the latter including the analysis of web-based searches to assess individuals’ health-seeking behavior [6]. Google Trends (GT) is one of the most frequently used tools to assess trends in web-based searches. This Google service displays the relative volume of searches for which a keyword (or set of keywords) is entered into the Google search engine [7]. Web-based searches on *asthma* and related terms have been assessed in previous studies [8,9]. However, GT for the search term *asthma* [7] only allows for the easy identification of large outbreaks (such as thunderstorm-induced asthma) [10] or media coverage–driven search peaks [11]. In fact, seasonal variations in pollen concentrations, which influence the occurrence of asthma exacerbations, are not reflected in GT for *asthma* [9]. However, to date, no study has examined the relationship between GT for *common cold*, the major risk factor for asthma exacerbations, and asthma hospitalizations.

### Objectives

In this study, we aim to assess whether asthma hospitalizations could be predicted by GT along with data from previous hospital admissions. To do so, we aim (1) to assess and discuss GT for *common cold* in different countries of the world, (2) to correlate GT for *common cold* and hospitalization data, and (3) to build models forecasting asthma hospitalizations for a period of 1 year (based on GT and past hospitalization data), correlating observed and predicted asthma hospitalizations.

## Methods

### Study Design

We conducted an infodemiology study to (1) correlate GT on rhinovirus-related search terms with asthma hospitalizations for a period of approximately 5 years (2012-2016) in Portugal, Spain, Finland, Norway, and Brazil and (2) assess whether such GT, along with previous admissions, were able to predict asthma hospitalizations. This study complies with the methodological framework of Mavragani and Ochoa [12].

### Queries and Data Sources for GT for Rhinovirus-Related Search Terms

GT topics are groups of search terms that concern the same concept, irrespective of their language [9,12-14]. This is particularly relevant, as there are countries with several words and idioms referring to the *common cold* (a paradigmatic example is Spain, a country with five official and co-official languages) as well as others with ambiguous words referring to the *common cold* (eg, English-speaking countries, in which *cold* has a double meaning). Assessing data from several countries, we found that *common cold* was listed as a *pseudo-influenza syndrome* topic (this topic was renamed as *common cold* at the time of manuscript submission).

We then retrieved country-level GT on rhinovirus-related search terms from January 1, 2012, to December 17, 2016, in Portugal, Spain, Finland, Norway, and Brazil. The countries were selected according to the possibility of having nationwide weekly asthma hospitalization data available for comparison. To provide a wider perspective, we plotted GT patterns for the *pseudo-influenza syndrome* topic, not only in these 5 countries, in which asthma hospitalizations were also assessed, but also in 11 additional countries.

The assessed time frame was selected considering both GT and asthma hospitalization data. On the one hand, for periods longer than 5 years, GT data are presented on a monthly level rather than on a weekly level (with monthly intervals being insufficiently sensitive for assessing variations in asthma hospitalizations). On the other hand, a complete period of 5 years was not assessed (with the last 2 weeks of December 2016 not being assessed) on account of available asthma hospitalization data (see the subsection *Asthma Hospitalization Data Sources*).

For each country, we tested two different GT queries: (1) one consisting of the *pseudo-influenza syndrome* topic, and (2) another being a combination of search terms consisting of words for *common cold* (selected on discussion with native speakers of each language). The search term combinations were as follows:

- Portugal: *constipação* + *resfriado*
- Spain: *resfriado* + *resfrío* + *catarro* + *constipado* + *refredat* + *constipate* + *arrefriado* + *hotzeri*
- Finland: *flunssa* + *nuha* + *vilustuminen*
- Norway: *forkjølelse* + *forkjøling* + *snue* + *krimsjuke*
- Brazil: *resfriado*

Quotation marks were not used because each keyword consisted of a single word. Misspellings or nonaccentuated forms were not included in the search term combinations. In Portugal, the words without diacritical marks (ie, *constipacao* or *constipaçao*) have a much lower relative volume of searches (with many *zero-value* observations) than the correct word *constipação*, with identical relative search volumes being observed whenever misspelled words are or are not included in search term combinations. In Spain, identical relative search volumes are also observed, whether or not the misspelled word *resfrio* is included in search term combinations. In Norway, the misspelled variants *forkjolelse* and *forkjoling* generate negligible results (ie, all observations with a relative search volume of zero).

State-level analyses were also performed in Spain and Brazil. For Spain, we performed separate analyses for the three most populous autonomous communities (Andalusia, Catalonia, and Madrid). For Brazil, we separately analyzed data from the most populous state in three of the five Brazilian geographical regions (as defined by the Brazilian Institute of Geography and Statistics): São Paulo (Southeast), Rio Grande do Sul (South), and Bahia (Northeast). For the two other geographical regions (North and Central-West), the most populous states had low-quality GT data (with many weeks recorded as 0).

GT was accessed via its web interface. Categories and subcategories were not selected in our searches. GT data sources, other than *web searches*, have not been used. Searches and data extraction were performed on January 13, 2020, with a single data extraction for each country.

### Asthma Hospitalization Data Sources

In the 5 studied countries, we assessed all hospitalizations with asthma as the main diagnosis (ie, International Classification of Diseases, Ninth Revision, Clinical Modification code 493.x or International Classification of Diseases, Tenth Revision, code J45) occurring in public hospitals from January 1, 2012, to December 17, 2016 (the last 2 weeks of 2016 were not included, as we did not have any information on 2017 Portuguese and Brazilian discharges, and many patients admitted at the end of 2016 were discharged in 2017). Hospitalization data were retrieved from (1) the Hospital Morbidity database (provided by the Portuguese Central Administration of the Healthcare System) for Portugal, (2) the Hospital Morbidity Survey databases (*Encuesta de morbilidad hospitalaria, Instituto Nacional de Estadistica*) for Spain, (3) the National Hospital Discharge Register (*Hoitoilmoitusrekisteri*, HILMO) for Finland, (4) the Norwegian Patient Registry (*Norsk Pasientregister*) for Norway, and (5) DATASUS data from the Single Health System (*Sistema Único de Saúde*) for Brazil.

### Statistical Analysis

In brief, we performed two major types of analyses. First, we assessed the correlations between GT data and asthma hospitalizations in each country after applying time series analysis methods. Subsequently, we built models forecasting asthma hospitalizations for a period of 1 year based on GT and hospitalization data from the previous 3 years. To test for the predictive ability of the models, forecasted and observed asthma admissions were compared. Both GT and hospitalization data were presented weekly. The performance of analysis on a weekly basis allowed for the detection of short-term variations without the large random fluctuations that can be observed when data are analyzed on a daily basis.

In detail, we calculated Pearson correlation coefficients to assess the correlation between GT data and asthma hospitalizations in each country. In addition, we performed cross-correlation analysis because (1) for GT data, a relevant trend is expected, mirroring an increase in Google searches with the passing of years, and (2) GT results are expressed as relative search values (ie, percentages in relation to the maximum observed value of the whole period), whereas hospitalizations are expressed as absolute values. The time series can be decomposed into three components: trend, seasonal effects, and random errors. We removed the trend component for both GT and hospitalization data and then estimated cross-correlation coefficients between GT and hospitalization data of the same week and with different week lags (namely, with 1, 2, 3, and 4 weeks of difference between GT data and hospitalization data, to assess whether search volumes displayed better correlation with asthma hospitalizations occurring in subsequent weeks than with those occurring in the same week).

We built seasonal autoregressive integrated moving average (ARIMA) models to forecast variations in asthma hospitalizations over a period of 1 year. We started in the Northern Hemisphere Summer of 2015 (week of June 21, 2015) and based our models on the trend component of asthma hospitalizations and on GT data from the 3 previous years (weeks of July 1, 2012-June 14, 2015). Seasonal ARIMA models are defined by the parameters (*p*, *d*, *q*)(*P*, *D*, *Q*)*_s_*, where *p* corresponds to the order of autoregression, *d* is the degree of difference, *q* is the order of the moving average part, *P* is the seasonal order of autoregression, *D* is the seasonal integration, *Q* is the seasonal moving average, and *s* is the length of the seasonal period [11,15]. We applied seasonal ARIMA (3,0,2)(0,1,1)_52_ models. Such models were chosen, among others with *p*=3 or *p*=4 (as suggested by the time series autocorrelation function plots) and with other parameters, on account of their lower corrected Akaike information criteria and nondetection of correlated residuals (both as assessed by the Ljung-Box test and by the autocorrelation function plots, with no significant spikes being observed). Asthma hospitalizations and GT data from July 2012 to June 2015 were used as a training set, with hospitalizations between the weeks of June 21, 2015, and June 19, 2016 being forecasted based on observed GT for that period. The predictive ability of the models was assessed by calculating (1) the correlation coefficients between the predicted variation in hospitalizations and the observed trend in hospitalizations for that period, (2) the correlation coefficients between the predicted variation in hospitalizations and the actual number of asthma hospitalizations (ie, without time series decomposition) for that period, (3) the average weekly difference between the numbers of predicted and observed hospitalizations, and (4) the number of weeks whose number of observed asthma hospitalizations fell outside the 95% CI for predicted admissions.

Normality was assessed from the skewness and kurtosis of each distribution (with values lower than −1 or higher than 1 indicating deviation from normality). The 95% CIs of the correlation coefficients were computed to assess their precision and determine whether they were significantly different from 0. All analyses were performed using the R software, version 4.0.0 (R Foundation for Statistical Computing).

## Results

### Overview

**Figure 1:**
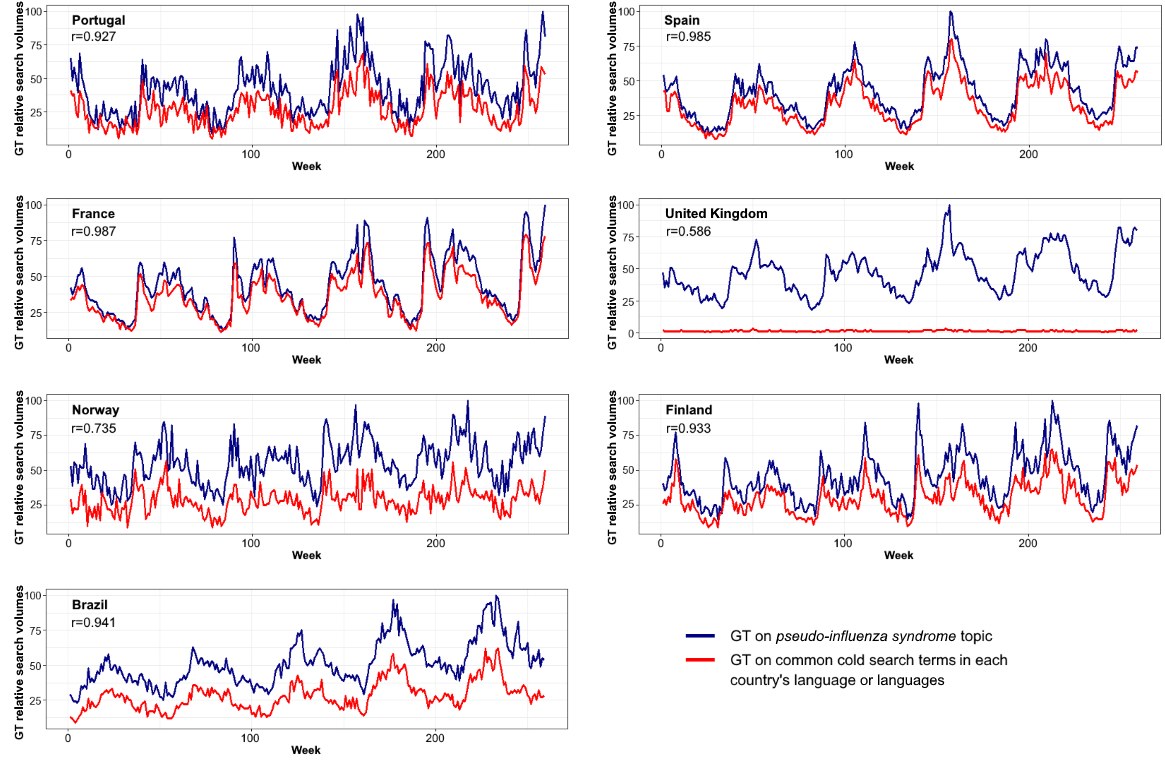
Google Trends data on the *pseudo-influenza syndrome* topic and on *common cold* search terms in each country’s respective language or languages (r: Pearson correlation coefficient). For the United Kingdom, r=0.769 when GT data on *pseudo-influenza syndrome* and on *common cold* search terms are retrieved separately. GT: Google Trends.

Assessing data from several countries, we observed that *common cold* was listed as *pseudo-influenza syndrome* topic, although with variable correlation between GT on the *pseudo-influenza syndrome* topic and *common cold* words (Figure 1). A relatively low correlation coefficient was observed for the United Kingdom, as we compared the *pseudo-influenza syndrome* topic with the search expression *common cold*, which may not be frequently used, particularly when compared with the term *cold*. However, we did not query the latter term because of its double meaning.

**Figure 2:**
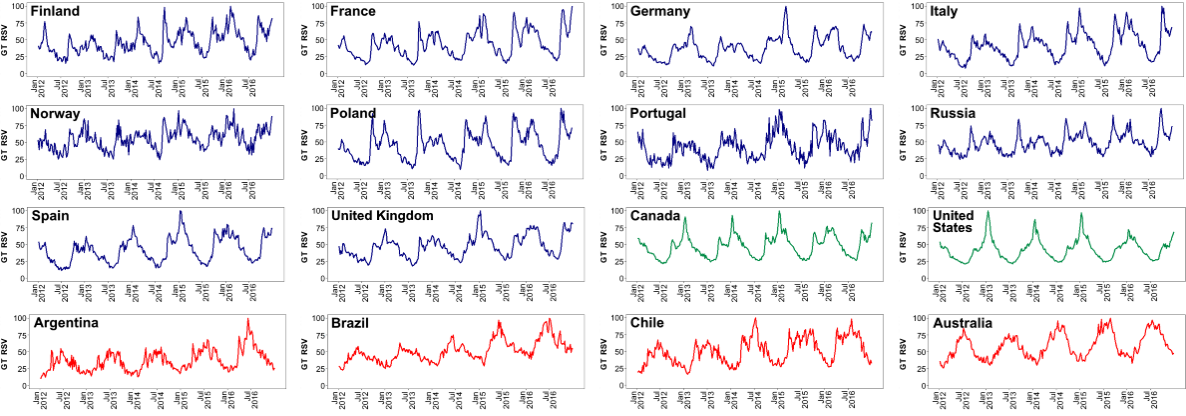
Google Trends data on *pseudo-influenza syndrome* for 16 countries in Europe (blue), North America (green), and the Southern Hemisphere (red) for a period of 5 years (2012-2016). GT: Google Trends; RSV: relative search volume.

Between 2012 and 2016, GT data for *pseudo-influenza syndrome* presented similar patterns across 16 countries for which GT data were plotted, with peaks in the winter and valleys in the summer (Figure 2). In the 5 main assessed countries, correlations between untransformed GT on the *pseudo-influenza syndrome* topic and asthma hospitalizations varied between 0.10 (in the Brazilian state of Bahia) and 0.69 (in Spain; Table 1). Similar values were observed when analyzing the correlations between GT and words for the common cold.

**Table 1:** Correlation and cross-correlation coefficients between common cold Google Trends data (ie, Google Trends data on the pseudo-influenza syndrome topic and on common cold search terms) and asthma hospitalizations for the period 2012-2016.

Country or region		Correlation coefficients (95% CI) based on observed data	Cross-correlation coefficients (95% CI) after removal of the trend component				
			Week lag^a^-0	Week lag^a^-1	Week lag^a^-2	Week lag^a^-3	Week lag^a^-4
***Pseudo-influenza syndrome* topic**							
	Portugal	0.54 (0.42 to 0.66)	0.68 (0.54 to 0.81)	0.73 (0.59 to 0.86)	0.67 (0.53 to 0.81)	0.71 (0.57 to 0.85)	0.65 (0.52 to 0.79)
	Spain	0.69 (0.57 to 0.81)	0.83 (0.69 to 0.97)	0.84 (0.71 to 0.98)	0.83 (0.69 to 0.96)	0.80 (0.67 to 0.94)	0.76 (0.62 to 0.89)
	Andalusia	0.54 (0.42 to 0.66)	0.63 (0.50 to 0.77)	0.68 (0.55 to 0.82)	0.68 (0.54 to 0.81)	0.67 (0.54 to 0.81)	0.66 (0.52 to 0.80)
	Catalonia	0.65 (0.53 to 0.78)	0.80 (0.66 to 0.93)	0.80 (0.66 to 0.93)	0.79 (0.65 to 0.93)	0.79 (0.65 to 0.92)	0.74 (0.60 to 0.87)
	Madrid	0.63 (0.51 to 0.75)	0.67 (0.53 to 0.80)	0.69 (0.55 to 0.82)	0.65 (0.52 to 0.79)	0.65 (0.52 to 0.79)	0.62 (0.48 to 0.76)
	Finland	0.16 (0.04 to 0.29)	0.44 (0.30 to 0.58)	0.44 (0.30 to 0.57)	0.42 (0.29 to 0.56)	0.32 (0.18 to 0.46)	0.25 (0.11 to 0.39)
	Norway	0.15 (0.03 to 0.27)	0.32 (0.18 to 0.45)	0.35 (0.21 to 0.49)	0.26 (0.12 to 0.39)	0.18 (0.05 to 0.32)	0.18 (0.05 to 0.32)
	Brazil	0.26 (0.14 to 0.39)	0.83 (0.69 to 0.97)	0.77 (0.64 to 0.91)	0.70 (0.57 to 0.84)	0.62 (0.49 to 0.76)	0.54 (0.40 to 0.67)
	São Paulo	0.45 (0.33 to 0.57)	0.66 (0.52 to 0.80)	0.58 (0.45 to 0.72)	0.44 (0.30 to 0.58)	0.33 (0.19 to 0.47)	0.28 (0.14 to 0.42)
	Rio Grande do Sul	0.54 (0.42 to 0.66)	0.67 (0.53 to 0.80)	0.64 (0.50 to 0.77)	0.59 (0.45 to 0.72)	0.57 (0.44 to 0.71)	0.53 (0.40 to 0.67)
	Bahia	0.10 (−0.02 to 0.22)	0.50 (0.37 to 0.64)	0.49 (0.36 to 0.63)	0.54 (0.40 to 0.68)	0.47 (0.33 to 0.61)	0.40 (0.26 to 0.53)
***Common cold* search terms**							
	Portugal	0.53 (0.41 to 0.65)	0.63 (0.50 to 0.77)	0.68 (0.54 to 0.82)	0.68 (0.54 to 0.81)	0.65 (0.51 to 0.79)	0.59 (0.45 to 0.72)
	Spain	0.69 (0.57 to 0.82)	0.82 (0.69 to 0.96)	0.84 (0.70 to 0.97)	0.82 (0.68 to 0.96)	0.80 (0.66 to 0.94)	0.75 (0.62 to 0.89)
	Andalusia	0.55 (0.43 to 0.67)	0.62 (0.48 to 0.76)	0.65 (0.51 to 0.78)	0.66 (0.53 to 0.80)	0.65 (0.52 to 0.79)	0.66 (0.52 to 0.79)
	Catalonia	0.67 (0.55 to 0.79)	0.78 (0.65 to 0.92)	0.78 (0.65 to 0.92)	0.78 (0.64 to 0.92)	0.76 (0.62 to 0.90)	0.71 (0.58 to 0.85)
	Madrid	0.61 (0.49 to 0.73)	0.63 (0.50 to 0.77)	0.66 (0.52 to 0.80)	0.64 (0.50 to 0.78)	0.64 (0.50 to 0.78)	0.62 (0.48 to 0.76)
	Finland	0.24 (0.12 to 0.36)	0.47 (0.34 to 0.61)	0.46 (0.32 to 0.59)	0.40 (0.26 to 0.54)	0.32 (0.19 to 0.46)	0.25 (0.11 to 0.38)
	Norway	0.22 (0.10 to 0.35)	0.35 (0.21 to 0.49)	0.33 (0.20 to 0.47)	0.24 (0.11 to 0.38)	0.15 (0.02 to 0.29)	0.15 (0.02 to 0.29)
	Brazil	0.37 (0.25 to 0.49)	0.82 (0.69 to 0.96)	0.77 (0.63 to 0.91)	0.69 (0.55 to 0.82)	0.61 (0.47 to 0.74)	0.52 (0.38 to 0.65)
	São Paulo	0.46 (0.34 to 0.58)	0.67 (0.54 to 0.81)	0.60 (0.47 to 0.74)	0.46 (0.33 to 0.60)	0.37 (0.24 to 0.51)	0.30 (0.16 to 0.43)
	Rio Grande do Sul	0.55 (0.43 to 0.67)	0.61 (0.48 to 0.75)	0.57 (0.43 to 0.70)	0.53 (0.40 to 0.67)	0.52 (0.38 to 0.65)	0.46 (0.33 to 0.60)
	Bahia	0.18 (0.06 to 0.30)	0.40 (0.26 to 0.54)	0.43 (0.30 to 0.57)	0.40 (0.26 to 0.54)	0.35 (0.21 to 0.48)	0.29 (0.15 to 0.43)

^a^Week lag corresponds to the week difference between Google Trends and hospitalization data (eg, a week lag of 1 implies that Google Trends data of a certain week will be correlated with hospitalization data of the following week).

### Time Series Results

**Figure 3:**
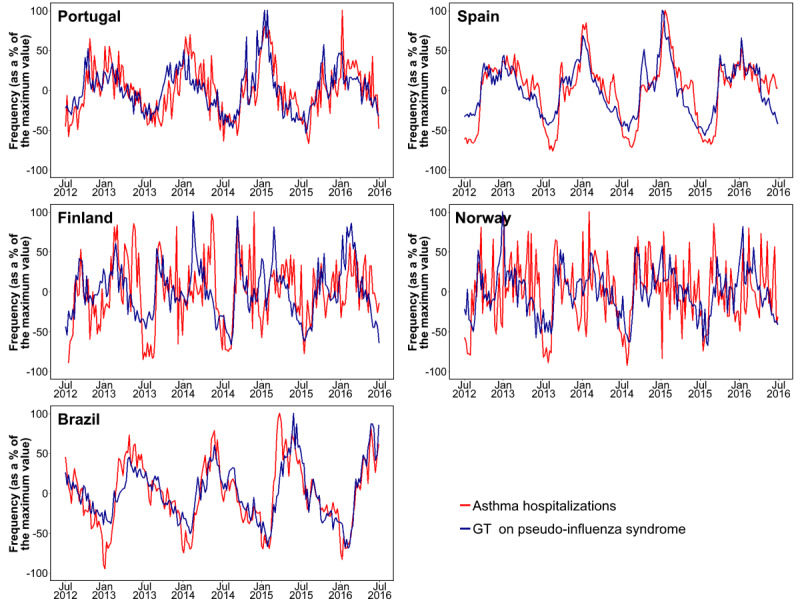
Google Trends data on *pseudo-influenza syndrome* and asthma hospitalizations (2012-2016) in Portugal, Spain, Finland, Norway, and Brazil. The trend component of time series has been plotted after removal of the seasonal effects and random error components. GT: Google Trends.

In time series analyses, GT on the *pseudo-influenza syndrome* topic correlated more strongly with asthma hospitalizations occurring in the subsequent week than with those occurring in the same week in Portugal (*ρ*=0.73 vs *ρ*=0.68), Spain (*ρ*=0.84 vs *ρ*=0.83), and Norway (*ρ*=0.35 vs *ρ*=0.32) but not in Finland (*ρ*=0.44 in both cases) or Brazil (*ρ*=0.77 vs *ρ*=0.83; Table 1; Figure 3). Similar results were observed with GT on *common cold* search terms (Portugal: *ρ*=0.68 vs *ρ*=0.63; Spain: *ρ*=0.84 vs *ρ*=0.82; Finland: *ρ*=0.47 vs *ρ*=0.46; Norway: *ρ*=0.32 vs *ρ*=0.35; Brazil: *ρ*=0.77 vs *ρ*=0.82). Relevant regional differences were observed in Spain and Brazil. In Spain, stronger correlations were observed in Catalonia than in Madrid or Andalusia. In Brazil, stronger correlations were observed in Rio Grande do Sul and São Paulo than in Bahia.

Forecasts for 1-year (June 2015 to June 2016) variations in asthma hospitalizations obtained through seasonal ARIMA models strongly correlated with actual observed asthma hospitalizations for the same period in Spain (*ρ*=0.88-0.91), Brazil (*ρ*=0.87-0.94), and Portugal (*ρ*=0.69-0.79; Table 2). Such correlations were moderate for Finland (*ρ*=0.49-0.55) and Norway (*ρ*=0.37-0.45). In Spain, the strongest correlations were observed for Catalonia (*ρ*=0.86-0.87), whereas in Brazil, they were observed for Rio Grande do Sul (*ρ*=0.89-0.91).

**Figure 4:**
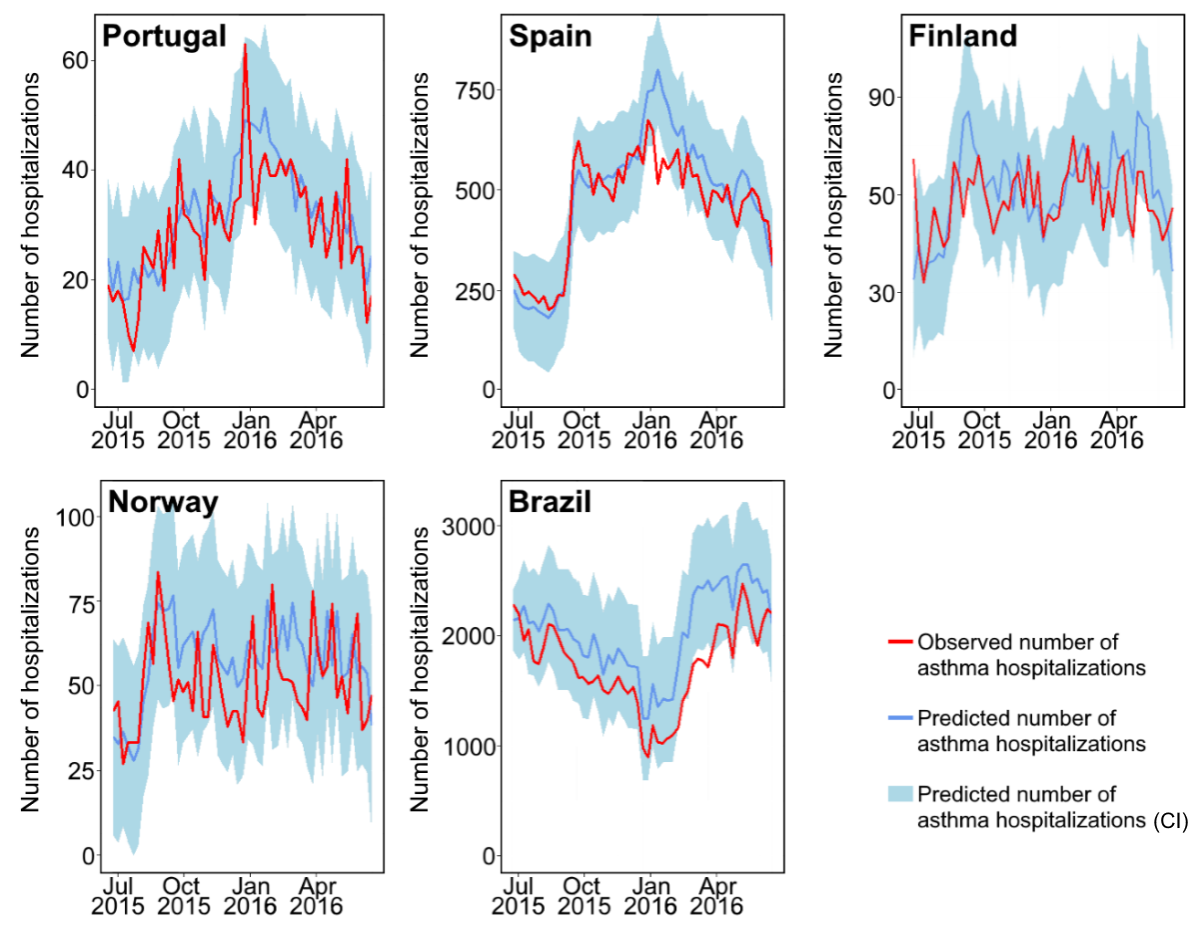
Predicted and observed number of asthma hospitalizations for 1 year in Portugal, Spain, Finland, Norway, and Brazil. Predicted hospitalizations were estimated based on previous hospitalizations and on Google Trends data for the *pseudo-influenza syndrome* topic.

**Figure 5:**
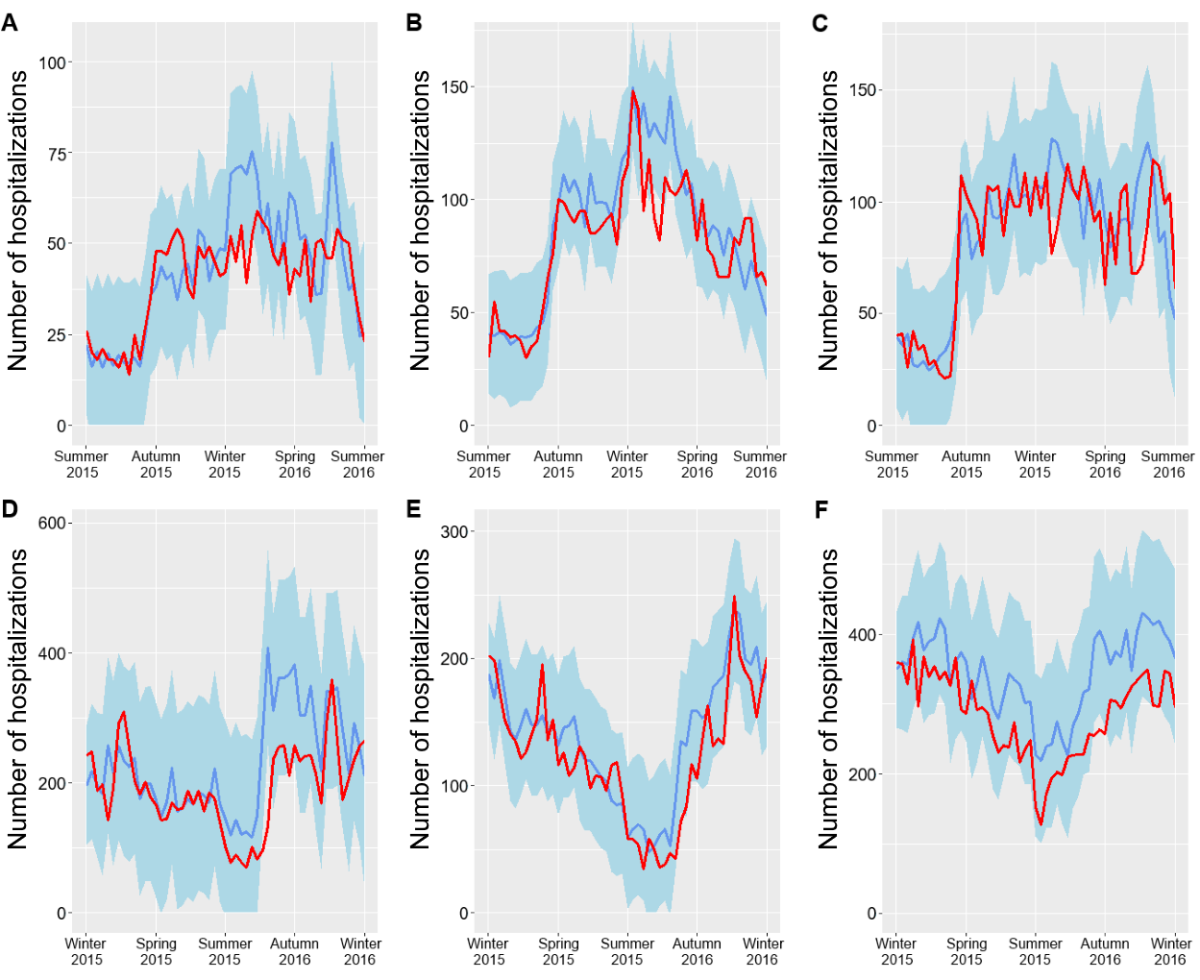
Predicted (blue; 95% CI in light blue) and observed (red) number of asthma hospitalizations for 1 year in the Spanish Autonomous Communities of Andalusia (A), Catalonia (B), and Madrid (C), as well as in the Brazilian States of São Paulo (D), Rio Grande do Sul (E), and Bahia (F).

From June 2015 to June 2016, we also forecasted the number of asthma hospitalizations occurring each week and compared it with the number of observed asthma hospitalizations (Table 3; Figures 4 and 5). The weekly number of predicted hospitalizations showed, on average, a 12% difference compared with the number of observed asthma hospitalizations in Spain. This difference was 23% in Portugal, 16%-17% in Finland, 32%-33% in Norway, and 21%-23% in Brazil. In 1 year, the number of weeks in which the absolute frequency of observed asthma hospitalizations did not fall within the predicted 95% CI ranged between 0 (Rio Grande do Sul) and 8 (Brazil as a whole).

**Table 2:** Results of forecasts for 1-year variations in asthma hospitalizations: correlation coefficients between predicted variations in asthma hospitalizations and actually observed asthma hospitalizations over 1 year (June 2015 to June 2016).

Country or region		Transformed observed hospitalizations^a^, correlation coefficient (95% CI)	Untransformed observed hospitalizations^b^, correlation coefficient (95% CI)
***Pseudo-influenza syndrome* topic**			
	Portugal	0.79 (0.67-0.88)	0.74 (0.60-0.84)
	Spain	0.90 (0.82-0.95)	0.88 (0.79-0.93)
	Andalusia	0.75 (0.61-0.85)	0.75 (0.60-0.85)
	Catalonia	0.87 (0.77-0.93)	0.86 (0.77-0.92)
	Madrid	0.83 (0.72-0.90)	0.82 (0.70-0.89)
	Finland	0.54 (0.26-0.73)	0.49 (0.19-0.71)
	Norway	0.37 (0.09-0.60)	0.41 (0.15-0.64)
	Brazil	0.93 (0.89-0.96)	0.87 (0.80-0.92)
	São Paulo	0.68 (0.45-0.84)	0.67 (0.51-0.79)
	Rio Grande do Sul	0.89 (0.83-0.94)	0.91 (0.86-0.95)
	Bahia	0.85 (0.74-0.92)	0.81 (0.72-0.89)
***Common cold* search terms**			
	Portugal	0.76 (0.63-0.85)	0.69 (0.58-0.79)
	Spain	0.91 (0.85-0.95)	0.88 (0.77-0.95)
	Andalusia	0.79 (0.67-0.87)	0.78 (0.64-0.88)
	Catalonia	0.87 (0.81-0.93)	0.86 (0.79-0.92)
	Madrid	0.84 (0.74-0.91)	0.83 (0.72-0.90)
	Finland	0.55 (0.26-0.75)	0.49 (0.18-0.72)
	Norway	0.39 (0.16-0.58)	0.45 (0.19-0.63)
	Brazil	0.94 (0.90-0.96)	0.88 (0.82-0.92)
	São Paulo	0.73 (0.50-0.88)	0.72 (0.59-0.81)
	Rio Grande do Sul	0.89 (0.82-0.94)	0.90 (0.84-0.95)
	Bahia	0.85 (0.77-0.92)	0.81 (0.71-0.89)

^a^Correlation coefficients between predicted weekly asthma hospitalization trends and actual observed hospitalizations after applying time series analysis methods (ie, after removing the trend component).

^b^Correlation coefficients between predicted weekly hospitalization trends and actual observed raw numbers of weekly asthma hospitalizations.

**Table 3:** Results of 1-year (June 2015-June 2016) forecasts for the number of asthma hospitalizations based on autoregressive integrated moving average models including common cold–related Google Trends data and asthma hospitalizations of the previous 3 years.

Country or region			Correlation (95% CIs) between number of predicted and observed hospitalizations		Average difference in the absolute numbers of predicted and observed weekly hospitalizations, N (average % difference)		Weeks with observed hospitalizations outside predicted 95% CIs, n (%)
***Pseudo-influenza syndrome* topic**							
	Portugal	0.79 (0.68-0.87)		5 (23.3)		1 (1.9)	
	Spain	0.92 (0.85-0.96)		54 (11.6)		4 (7.5)	
	Andalusia	0.76 (0.62-0.85)		9 (21.5)		5 (9.4)	
	Catalonia	0.88 (0.80-0.92)		13 (16.3)		5 (9.4)	
	Madrid	0.80 (0.67-0.88)		15 (21.1)		6 (11.3)	
	Finland	0.45 (0.16-0.69)		10 (16.7)		4 (7.5)	
	Norway	0.40 (0.17-0.59)		14 (31.8)		1 (1.9)	
	Brazil	0.88 (0.80-0.92)		328 (20.6)		7 (13.2)	
	São Paulo	0.63 (0.40-0.80)		52 (32.3)		3 (5.7)	
	Rio Grande do Sul	0.88 (0.82-0.93)		22 (22.8)		1 (1.9)	
	Bahia	0.79 (0.67-0.87)		63 (24.4)		5 (9.4)	
***Common cold* search terms**							
	Portugal	0.77 (0.65-0.86)		6 (22.5)		1 (1.9)	
	Spain	0.90 (0.85-0.96)		54 (11.6)		4 (7.5)	
	Andalusia	0.78 (0.66-0.88)		9 (23.5)		6 (11.3)	
	Catalonia	0.86 (0.75-0.92)		13 (16.1)		6 (11.3)	
	Madrid	0.81 (0.68-0.90)		15 (21.4)		7 (13.2)	
	Finland	0.47 (0.24-0.65)		9 (15.8)		3 (5.7)	
	Norway	0.40 (0.14-0.59)		15 (32.8)		3 (5.7)	
	Brazil	0.94 (0.90-0.96)		359 (22.6)		8 (15.1)	
	São Paulo	0.68 (0.42-0.83)		48 (30)		2 (3.8)	
	Rio Grande do Sul	0.89 (0.84-0.94)		19 (20)		0 (0)	
	Bahia	0.85 (0.77-0.92)		63 (24.3)		5 (9.4)	

## Discussion

### Principal Findings

In this study, we observed moderate-to-strong correlations between rhinovirus-related GT and asthma hospitalization data in 5 different countries (Portugal, Spain, Finland, Norway, and Brazil). In addition, based on previous admission patterns and rhinovirus GT, we built seasonal ARIMA models with good capacity to forecast asthma hospitalizations.

Although the overall observed correlations were moderate to strong, and the forecast models showed good performance, between-country differences should be highlighted. Overall, lower correlations between rhinovirus-related GT and hospitalization data were observed in Finland and Norway. In these 2 countries, the analysis of our data indicates that asthma hospitalizations have markedly decreased throughout the years. Such a decrease may be partly explained by a focus on the early detection and prevention of exacerbations. Such goals are indeed stated in the Finnish Asthma Programme 1994-2004 and in the Finnish Allergy Programme 2008-2018, which have both promoted guided self-management (ie, patients’ identification of causes and proactive prevention of exacerbations) as the primary form of treatment [16,17]. This better overall asthma control (with a more effective prevention of respiratory infection–related exacerbations) may in part be explained by the lower correlations between common cold–related GT and asthma hospitalizations. On the other hand, differences between Finland and Norway may be explained by the more frequent fluctuations in Norwegian asthma hospitalizations (even within the same season), which can be explained by geographic or climatic reasons: Norway has a long coast along several seas in the Atlantic and Arctic with more than five Köppen climate zones, whereas Finland only lies in the Baltic Sea with three Köppen climate zones. In fact, for Norway, a stronger cross-correlation (0.46) was observed when performing analyses based on 15-day average values instead of weekly values. These frequent fluctuations also explain why, despite the moderate correlations, the observed number of Norwegian hospitalizations fell within the forecasted 95% CIs in 49-51 out of 52 weeks (June 2015-June 2016).

On the other hand, within-country differences should also be considered. For example, in Spain, the strongest correlations between observed and predicted asthma hospitalizations were related to the autonomous community of Catalonia. The fact that correlations were weaker in other areas may be partly explained by the seasonal asthma hospitalization peaks observed in Madrid and Andalusia, which had no correspondence with rhinovirus-related GT data. In fact, such peaks correlate with olive pollen peaks, and olive pollen concentrations are much higher in Madrid and Andalusia than in Catalonia [18]. Differences in pollen exposure may also partly explain the worse results observed in Portugal (when compared with Spain), where olive trees are abundant throughout all inland regions. In Brazil, the best results were consistently observed for Rio Grande do Sul, a state with only two Köppen climate zones, compared with seven in São Paulo and nine in Bahia. Therefore, although rhinovirus-related GT, along with previous hospitalization patterns, may help to forecast trends in asthma hospitalizations, improved accuracy is to be expected if other variables are also taken into account. Such variables may include pollen exposure, pollution levels, and even meteorological variables. This is in line with the multifactorial nature of asthma exacerbations, in which viral infections play a major role along with other environmental factors.

### Strengths and Limitations

**Figure 6:**
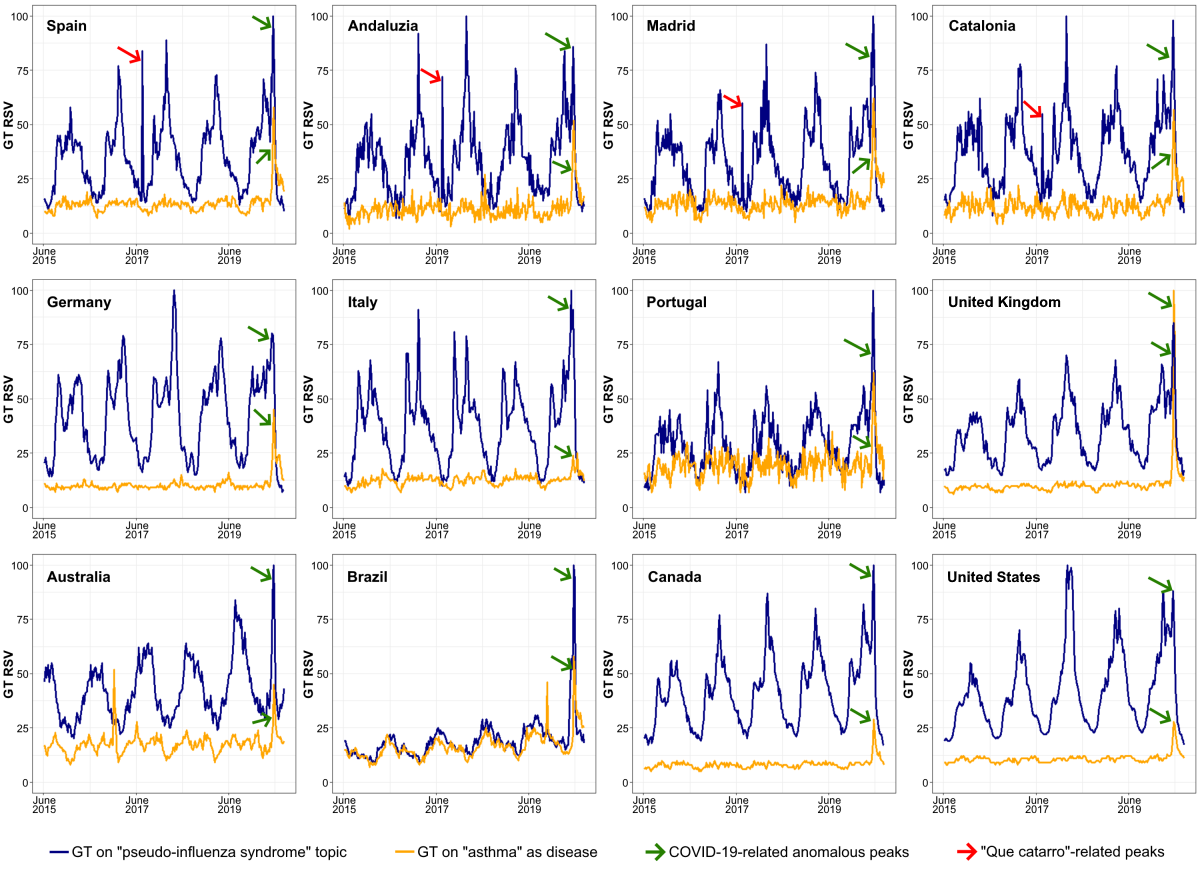
*Pseudo-influenza syndrome* Google Trends data for the period 2015-2020, with anomalous peaks evidenced (anomalous peaks associated with COVID-19: green arrows; peaks associated with transmission of the cartoon *Que catarro* on Spanish television in June 2017: red arrows). GT: Google Trends; RSV: relative search volume.

This study had several important limitations. First, GT provides information on users’ search behaviors, which reflect not only the true epidemiological situation of a disease or condition but also other factors, such as media coverage or users’ interest or curiosity [6]. Such factors are particularly evident when assessing anomalous peaks in pseudo-influenza GT data over the last 5 years (2015-2020). For example, in all regions of Spain (Figure 6; red arrows), but not in other countries, there was a 1-day peak at the end of June 2017. This peak was probably related to the TV cartoon episode *Qué catarro*, which was aired at that time across Spain [19,20] (*catarro* is a Spanish or Castilian word for *common cold*). In another example, a larger anomalous pseudo-influenza GT data peak was found in several countries during the COVID-19 outbreak in 2020 (Figure 6; green arrows). Previous studies have shown that GT can be highly influenced by media attention. For example, for several countries in Europe and America, search peaks on anosmia or ageusia had a better correlation with media coverage than with the epidemiological situation of COVID-19 [21]. Media coverage also appears to have played a decisive role in driving search peaks for asthma during the COVID-19 pandemic [11]. Although, at first sight, we could believe that media coverage does not particularly bias GT data in this study (as the common cold is a frequent and mild condition that does not drive media attention), the previously discussed Spanish anomalous data peak suggests that this factor should not be discarded.

Additional limitations include the absence of GT data on the actual number of performed searches, with only normalized data provided (ie, data expressed as a percentage of the search volume in the period during which the term or expression gathered the most attention). In states or regions with a low volume of searches, the quality of the data provided by GT is not sufficient for analysis. As a result, we were not able to assess all Brazilian states (not even the most populous Brazilian state in every region) nor all Spanish autonomous communities. Nevertheless, we assessed the most populous Brazilian state in three of the five official geographical regions, as well as the three most populous Spanish autonomous communities. Another limitation concerns the secular increase in the use of the internet (and therefore of Google searching) between 2012 and 2016, particularly with the generalization of smartphone use. The share of searches using Google may also vary during this period. In Brazil, Google was responsible for 85% of the searches performed in 2013, but this percentage increased to over 95% in 2015 [22,23]. To control these secular trends, we applied time series analysis methods, removing the estimated trend components for both GT and hospitalization data.

Finally, in this study, we solely considered asthma hospitalizations, even though only a minority of exacerbations resulted in hospitalization. Although trends in hospitalizations probably mirror those in exacerbations, an assessment of both hospitalizations and emergency department visits (whose data were not currently available for analysis) would probably more accurately reflect trends in exacerbations. Furthermore, the generalizability of our forecast models is limited, as such models require previous hospitalization data, which may not be easily available in all countries.

This study had several strengths. In particular, we assessed 5 different countries (4 in Europe and 1 in South America) using nationwide data for a period of 5 years. For 2 of these countries, we considered regional differences to assess state-level data. In addition, rhinovirus-related GT data were retrieved by two different strategies—GT data on the *pseudo-influenza syndrome* topic and on search terms regarding the common cold—which obtained comparable results. Finally, for data analysis, we applied methods to remove secular trends of GT data and hospitalizations and built seasonal ARIMA models with predictions of hospitalization trends and frequencies that had similar results.

### Conclusions

In this study, we found that rhinovirus-related GT data correlated well with asthma hospitalizations in Portugal, Spain, and Brazil (with moderate correlations observed for Finland and Norway). In addition, such GT data, along with previous admission patterns, were able to reasonably forecast asthma hospitalizations in the examined countries. Although these results suggest that rhinovirus-related GT data may be helpful when building models to predict asthma hospitalizations, future studies should explore the design of more complex models, taking environmental variables into account and possibly assessing exacerbations or other outcome variables.

## Abbreviations

ARIMA: autoregressive integrated moving average
GT: Google Trends
